# Could radiological parameters help to predict the failure of ureteral access sheath placement

**DOI:** 10.1007/s00240-024-01599-8

**Published:** 2024-06-25

**Authors:** Erhan Erdogan, Taha Yusuf Kuzan, Emre Burak Sahinler, Ahmet Fatih Kanberoglu, Mehmet Uslu, Ozgur Arikan, Resul Sobay, Alper Asik, Kemal Sarica

**Affiliations:** 1 Department of Urology, Sancaktepe Sehit Prof. Dr. Ilhan Varank Research and Training Hospital, Istanbul, Turkey; 2 Department of Urology, Health Practice and Research Hospital, Kafkas University, Kars, Turkey; 3 Department of Urology, Istanbul Medeniyet University Goztepe Suleyman Yalcin City Hospital, Istanbul, Turkey; 4 Department of Urology, Umraniye Research and Training Hospital, Istanbul, Turkey; 5 Department of Urology, Medical School, Biruni University, Istanbul, Turkey

**Keywords:** Ureteral Access Sheath, Retrograde Intrarenal surgery, Kidney ureter bladder (KUB)

## Abstract

To identify the radiological parameters which may help to predict the success of ureteral access sheath (UAS) placement during retrograde intrarenal surgery (RIRS).The study included 49 patients in whom failure ureteral access sheath placement in RIRS and 49 control group patients who were successfully placement between January 2023 and December 2023. The age, gender, body mass index (BMI), non-contrast computed tomography (NCCT), and kidney ureter bladder (KUB) radiographs were compared between the two groups. Measurements of the anteroposterior (ap) diameter of the pelvic inlet, anteroposterior diameter of the pelvic outlet, interspinous distance diameter were taken from non-contrast computed tomography (NCCT), while pelvic anteroposterior diameter and pelvic lateral diameter were measured from kidney ureter bladder (KUB) radiography. There were no significant differences between the groups in age, gender, body mass index, ap pelvic inlet diameter, ap pelvic outlet, and interspinous distance diameter. However, a statistically significant difference was found between the pelvic ap diameter and pelvic lateral diameter values measured on the KUB radiography. The values for pelvic ap diameter and pelvic lateral diameter measured in the KUB radiographs can be used to predict the likelihood of UAS passage during RIRC procedures. However, further studies with larger patient groups are needed to establish a cut-off value.

## Introduction

Currently endoscopic (ureteroscopic) management of both ureteral and renal stones is the most commonly preferred method due to the minimal invasive nature of the technique. Related with this issue, based on the remarkable advances in instrument technology and increasing experience, retrograde intrarenal surgery (RIRS) has gained world-wide popularity with its successful and safe outcomes in the renal stones up to 20 mm [1]. As an essential tool in RIRS management of upper tract stones, ureteral access sheaths (UAS) have been commonly used in an attempt to provide an effective drainage along with the reduction of intrarenal pressure for a safe procedure. Additionally, use of UAS provided increased visibility, reduced operative duration and allow multiple re-entries into the involved ureter without difficulty [2]. Although placement of UAS seems to be possible in the majority of the cases problems in its insertion has been reported in around 22% of patients planned for this procedure. Additionally, failure to perform a ureteroscopic procedure due to a tight (difficult) ureter not allowing a passage in around 8–10% of the cases [3]. In these cases, placement of a ureteral stent and postponement of the procedure for a second stage has been accepted as the standard approach. Related with this issue, although presence of a ureteral stent at first presentation and stent placement prior to RIRS procedure may dilate the and ease the procedure, as stent placement will require a second stage, this approach will not be practical in clinical practice [3, 4]. Thus, it may be of value and helpful if endourologists could identify the cases who need presenting to overcome all these difficulties.

In attempt to predict the likelihood of a smooth UAS insertion, some certain parameters like age, previous same-side procedures and presence of a preoperative stent were found to be independent factors on this aspect [3]. Preoperative presence of a stent in situ has been found to be highly predictive as expected [5]. In some other studies, while UAS placement was found to be high likely in patients with normal BMIs and a tent-shaped ureteral orifice [6], male gender and ipsilateral hydronephrosis have been reported to be associated with increased failure rates [7]. Based on all these clinically contradictory outcomes reported in highly limited publications, prediction of a successful UAS placement could give certain advantages for the practising urologists in order to outline their policy prior to RIRC procedures. Radiologic evaluation of stone and anatomy related factors may provide some insights on this aspect and noncontrast-enhanced computed tomography (NCCT) has been used in some trials to identify only the likelihood of ureteral stone passage but not the UAS placement [8–10]. No study so far did focused on the possible role of radiologic parameters in the prediction of UAS passage, and to the best of our knowledge, this study is the first report using radiologic NCCT and KUB measurement parameters on this aspect.

## Patients and methods

Medical records of 98 patients undergoing retrograde intrarenal surgery (RIRC) (for stones < 20 mm) between January 2023 and December 2023 were evaluated in a retrospective manner and cases were included into the study program. The study protocol was approved by the local ethical committee of the institution. Patients with a history of previous ureteral stenosis, ureteral operations, ureteral orifice abnormalities, urinary tract infection, any medication that may affect ureteral tonus/peristalsis and urogenital anomalies were all excluded from the study protocol.

Based on the success status of the success during UAS insertion patients were divided into two groups: Group 1 (n: 49) patients in whom a UAS could not be inserted successfully during the procedure and Group 2 (n: 49) cases in whom a UAS insertion was successful without any difficulty. Placement of UAS prior to RIRC procedure was performed under general anaesthesia in the lithotomy position. Following a retrograde pyelography evaluation and placement of a 0.038” guide wire into the ureter, a 9.5/11.5 Fr 45 cm coaxial UAS (GEOTEK Brand) was intended to be inserted into the ureter under fluoroscopic guidance. While the procedure has been performed in a standard manner after UAS placement, a double JJ stent was placed into the involved ureter in cases the placement of UAS was not possible. The procedure was planned for a second stage following the expected passive dilation of the ureter.

**Fig. 1:**
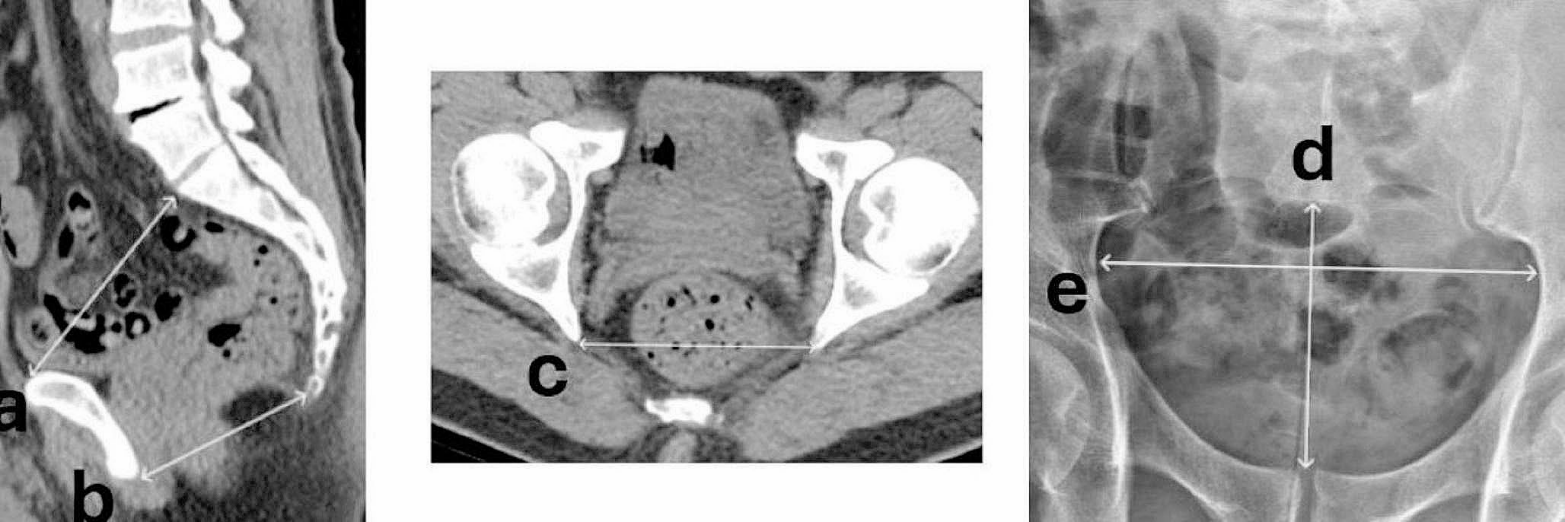
Measurement parameters in NCCT and KUB. **(a)** Anteroposterior diameter of the pelvic inlet. **(b)** Anteroposterior diameter of the pelvic outlet. **(c)** Interspinous diameter of the mid-pelvis. **(d)** Pelvic anteroposterior diameter. **(e)** Pelvic lateral diameter

In all cases some certain radiological parameters measured on both CT (antero-posterior (ap) pelvic inlet diameter, ap pelvic outlet diameter, interspinous distance) and on Kidney-Bladder-Ureter (KUB) (pelvic ap diameter and pelvic lateral diameter) were well evaluated and interpreted to predict the likelihood of UAS passage in these cases (Fig. 1).

For statistical analysis, the mean and standard deviation (SD) values were calculated in each group. With this aim Independent T test and Mann-Whitney U test were used to evaluate differences between two groups. For categorical parameters however, the Chi-square test has been used. A value of *p* < 0.05 was regarded to be statistically significant.

## Results

A total of 98 patients (Male: 69 and Female: 29 M/F: 2.3) were included and the mean age of the patients in overall group was 47 ± 12 years. While the mean age of the cases was 48 ± 13 years in Group 1, this value was 47 ± 11 years in Group 2. The mean body mass index ( BMI) values were 26.7 ± 3.3 and 26.8 ± 3.2 in Group 1 and 2 respectively. There were no significant differences regarding the age, gender and BMI values between the two groups (Table 1).

**Table 1:** Patient characteristics of all patients

		Failed Access	Successfully Access	*P* value
Total (n)		49	49	
Gender	Male/female	33/16	36/13	0.506
Age (mean ± SD)		48 ± 13	47 ± 11	0.663
BMI (mean ± SD)		26.7 ± 3.3	26.8 ± 3.2	0.909

Evaluation of the radiological parameters assessed and evaluated revealed that there was no statistically significant difference withrespect to the ap pelvic inlet diameter, ap pelvic outlet diameter, and interspinous distance values on NCCT images between the two groups (*p* = 0.2903, 0.3577 and 0.6150). However a statistically significant difference was found between the two groups regarding the pelvis ap diameter and pelvis lateral diameter values measured on KUB. (*p* = 0.0001 and 0.0028) (Table 2).

**Table 2:** Radiologic parameters of all patients

	Parameters	Failed Access	Successfully Access	*P* value
		(mm) (means ± SD)	(mm) (means ± SD)	
NCCT	ap pelvic inlet diameter	111 ± 9	114 ± 12	0.2903
	ap pelvic outlet diameter	91 ± 10	89 ± 10	0.3577
	Interspinosus distance	98 ± 10	97 ± 11	0.6150
KUB	Pelvis ap diameter	102 ± 14	116 ± 19	0.0001
	Pelvis lateral diameter	137 ± 19	148 ± 13	0.0028

## Discussion

Remarkable advances in instrument technology and increasing experience have caused significant changes in the treatment concepts of urinary stones. Regarding this issue, as a minimal invasive technique, flexible ureteroscopic stone disintegration has gained world-wide popularity with its successful and safe outcomes in the management renal stones up to 20 mm [1]. As an accessory tool in the successful performance of RIRS ureteral access sheaths (UAS) are being commonly used to provide an effective drainage and reduce the intrarenal pressure levels for a safe procedure. Apart from these advantages, published data so far have clearly demonstrated that the use of UAS could increase the visibility, reduce operative duration and allow multiple re-entries into the involved ureters [2]. Although its use seems to be associated with above mentioned critical advantages however, placement of UAS may not to be possible in around 22% of patients planned for this procedure. Additionally, failure to perform a ureteroscopic procedure due to a tight (difficult) ureter not allowing a passage in around 8–10% of the cases [3]. In case of such a difficulty during the procedure, placement of a ureteral stent and postponement of the procedure for a second stage has been accepted as the most rational approach. Related with this issue, presence of a ureteral stent in the involved reno-ureteral unit or placement of a stent prior to RIRS procedure have were found to dilate the ureter the and ease the procedure. However, as stent placement will require a second procedure for removal, this approach will not be rational and acceptable for the cases in clinical practice [3, 4]. Thus, it may be of value and helpful if endourologists could identify the cases who need presenting to overcome all these difficulties.

Taking the possible difficulty in the placement of an UAS during RIRS into account, physicians looked for some certain parameters to predict the likelihood of a smooth UAS insertion, and age, previous same-side procedures and presence of a preoperative stent were found to be independent factors on this aspect [3]. Presence of a ureteral stent in the involved reno-ureteral unit was reported to helpful and predictive [5]. Related with this issue again, although UAS placement was performed without any difficulty in patients with normal BMIs and in cases with a tent-shaped ureteral orifice [6], some other factors namely male gender and ipsilateral hydronephrosis were found to be associated with increased failure rates [7]. However, the published data so far on the predictive factors in the prediction of UAS passage are highly limited and contradictory. Studies evaluating the placement of UAS before RIRS procedures have pointed out that successful insertion of a UAS over the guidewire may not be possible in all cases with failure rates varying 10–22% [2–4]. As an advantage on this aspect the insertion of a ureteral stent before UAS placement was found to cause passive dilation and increase the success rates [3, 10]. According to one report however, UAS placement can also be unsuccessful in 7.7% of the cases even after ureteral stenting for a reasonable time period [11]. Taking the fact that routine ureteral stenting prior to UAS is not recommended by the European Association of Urology (EAU) guidelines [6] into account, it may be of great value to determine the risk factors in order to minimize the failure rates of UAS placement. In their original study, Alkhamees M et al. were able to show that none of the factors related to patient demographics or stone characteristics may be predictive enough for the failure of UAS placement [2]. On the other hand, as another diagnostic tool performed in all cases, radiologic evaluation of stone and anatomy related factors on this aspect may provide some valuable insights. Related with this issue, although non-contrast computed tomography (NCCT) has been used to identify the likelihood of ureteral stone passage [8–10], no study so far did focus on the possible role of radiologic parameters in the prediction of UAS passage, and to the best of our knowledge, this study is the first report using radiologic NCCT and KUB measurement parameters on this aspect.

In this present study we aimed to evaluate the possible predictive role of some demographic as well as radiological parameters identified in both KUB and NCCT images on the likelihood of UAS passage prior to RIRS procedures. Our findings demonstrated no predictive parameter could be assessed and used on this aspect with respect to the factors evaluated for both patient characteristics and NCCT measurements. However, pelvic ap diameter and pelvic lateral diameter measurement values on KUB images were found to be shorter in KUB images and the evaluation of these radiological factors could be helpful in the successful prediction of the likelihood of UAS placement. Further studies to support our findings and look for other radiologic parameters are certainly needed to identify reliable predictive factors on this aspect.

It is clear that prediction of a successful UAS placement could provide certain advantages for the practising urologists in order to outline their policy prior to RIRC procedures. This information will help to the urologist to make decision making well by informing the patient about the possible additional procedures. This will also lower the total cost of the procedures by limiting the use of UAS for a failed attempt.

## Conclusions

Taking the risk of unsuccessful UAS placement before RIRS procedures in certain percent of the cases into account, identification of reliable radiological factors to predict the likelihood of UAS placement is of greater importance. Measurement of pelvic ap diameter and pelvic lateral diameter on KUB radiographs scheduled seemed to predict the failure of UAS placement well in our study. Urologists will be able to make decision making well by informing the patient about the possible additional procedures and the total cost of the procedures will certainly be limited due to the lack of failed UAS attempt.

## Data Availability

No datasets were generated or analysed during the current study.
